# Visual discrimination and amodal completion in zebrafish

**DOI:** 10.1371/journal.pone.0264127

**Published:** 2022-03-02

**Authors:** Valeria Anna Sovrano, Sofia Vicidomini, Davide Potrich, Maria Elena Miletto Petrazzini, Greta Baratti, Orsola Rosa-Salva

**Affiliations:** 1 Center for Mind/Brain Sciences, University of Trento, Rovereto, Italy; 2 Department of Psychology and Cognitive Science, University of Trento, Rovereto, Italy; 3 School of Natural Sciences, University of Torino, Torino, Italy; 4 Department of Biomedical Sciences, University of Padova, Padova, Italy; Tokai University, JAPAN

## Abstract

While zebrafish represent an important model for the study of the visual system, visual perception in this species is still less investigated than in other teleost fish. In this work, we validated for zebrafish two versions of a visual discrimination learning task, which is based on the motivation to reach food and companions. Using this task, we investigated zebrafish ability to discriminate between two different shape pairs (i.e., disk vs. cross and full vs. amputated disk). Once zebrafish were successfully trained to discriminate a full from an amputated disk, we also tested their ability to visually complete partially occluded objects (amodal completion). After training, animals were presented with two amputated disks. In these test stimuli, another shape was either exactly juxtaposed or only placed close to the missing sectors of the disk. Only the former stimulus should elicit amodal completion. In human observers, this stimulus causes the impression that the other shape is occluding the missing sector of the disk, which is thus perceived as a complete, although partially hidden, disk. In line with our predictions, fish reinforced on the full disk chose the stimulus eliciting amodal completion, while fish reinforced on the amputated disk chose the other stimulus. This represents the first demonstration of amodal completion perception in zebrafish. Moreover, our results also indicated that a specific shape pair (disk vs. cross) might be particularly difficult to discriminate for this species, confirming previous reports obtained with different procedures.

## Introduction

Zebrafish represent one of the main animal models for recent neurobiological research, thanks to its suitability for developmental and molecular biology techniques, as well as for genetic manipulations (e.g., [1–5]). Moreover, visual sensory mechanisms have been relatively well-investigated in zebrafish and this species has been established as a standard model for the study of the visual system (e.g., [6]). Zebrafish can thus provide an invaluable tool for the investigation of the neural mechanisms of visual cognition. In comparison to the widespread use of zebrafish in neurobiology research, however, the perceptual and cognitive abilities of this species have been less investigated. Indeed, zebrafish cognitive abilities have been less explored than those of other teleosts, for which more varied and complex cognitive tasks are often available (e.g., [7–10]). The aim of the current work was thus to investigate, in zebrafish, two fundamental mechanisms at the basis of many perceptual and cognitive adaptations for visual processing: namely, the visual discrimination of different shapes and the amodal completion of partially occluded objects. Shape discrimination is, obviously, an important aspect of visual processing, involved in several visually guided behaviours and higher cognitive functions. Likewise, amodal completion, which allows the perception of partially occluded objects as completing behind the occluder, is crucial for successful adaptation to the visual environment [11]. In fact, in real-world visual scenes, the retinal projections of the objects present in the external environment can often overlap. In this case, the objects closer to the observer visually occlude parts of the objects located further away. Amodal completion mechanisms allow the perception of a partially occluded object as a whole entity, segregated from the background in its integrity. Amodal completion is the product of the interpolation and grouping mechanisms that the visual system uses to integrate sensory information. These mechanisms integrate fragmentary visual information, allowing to recognize an object despite the absence of any visual stimulation from its covered parts. The result of these processes is a unified representation of objects as a whole and segregated from the background. This is essential for visually guided foraging, as well as for the visual identification of predators and conspecifics. Amodal completion mechanisms are thus likely to support multiple behavioural functions critical for survival.

In most cases, this process also allows the observer to accurately represent the full shape of the partially occluded object. Amodal completion mechanisms thus avoid the misrepresentation of shape information due to fact that parts of the object are not visible, which alters the outline of the object’s retinal projection. Thus, amodal completion mechanisms are likely to support shape discrimination in real-world visual scenes.

Amodal completion has been demonstrated in a number of species ranging from human infants [12] to non-human primates [13, 14], rodents [15], birds [16, 17] and invertebrates [18], indicating the importance of the subtending mechanisms for animals’ adaptation. Among fish species, amodal completion has been first found in *Xenotoca eiseni* [19], and then shown in coral reef fish and even in sharks [20, 21].

The perceptual abilities of zebrafish have been well investigated for motion processing (e.g., [22–24]). This revealed the importance of motion cues for the social responses of this species (e.g., [25, 26]; see also [27] for a review on zebrafish social responses). Most studies on zebrafish motion perception took advantage of the spontaneous responses elicited by motion and social stimuli (e.g., optokinetic or optomotor responses and shoaling behaviours; see also [28–31], for the use of spontaneous responses to test episodic-like memory and numerical cognition). However, it has been shown that this species is also amenable to training either in the form of aversive learning (e.g., [32–34]) or by means of positive reinforcement (e.g., [35]). Studies using positive reinforcements (e.g., food or conspecifics) have revealed, for example, zebrafish abilities in colour and pattern discrimination [36–39]), as well as their more sophisticated cognitive skills, such as processing of ordinal numerical [40] and spatial information (e.g., [41–43]; see [44] for a review).

In zebrafish, colour discrimination has been more extensively tested than shape discrimination, which is sometimes considered a more complex task [39]. A recent study directly compared colour- and shape-discrimination abilities in zebrafish, using an automated conditioning chamber [39]. In this set-up, the animals were food reinforced for approaching one of the two stimuli that had to be discriminated (appetitive conditioning). For both shape- and colour-discrimination alike, zebrafish performance depended on the overall size (salience) of the stimuli. However, even when larger stimuli were used, zebrafish were not as good in discriminating shapes (experiment 4, large shape condition) as in discriminating colour plates (experiment 1, large discrimination condition). This suggests that shape discrimination might be particularly challenging for zebrafish [39]. In line with the results of [39], in a similar appetitive conditioning task, Agrillo et al. [45] reported that zebrafish were unable to discriminate two simple shapes (filled triangle vs. empty circle), even though fish of another species (redtail splitfin) trained with the same procedure, successfully solved the task. However, using a different appetitive conditioning task and small stimuli, Santacà et al. [46] revealed a similar performance both for shape and for colour discrimination. This strongly suggests that zebrafish performance could be extremely sensitive to minute variations in the task procedure as well as in the stimuli.

Zebrafish ability to discriminate between different shapes has been also studied using object-recognition or place-recognition tasks, that exploit fish exploratory responses to novel objects or locations (e.g., [47–49]). Interestingly, in some object recognition tasks, zebrafish larvae showed a superior performance in shape than in colour discrimination tasks, confirming that these effects could be both age- and task-dependent [50].

Moreover, in spontaneous object-recognition tasks, not all shape pairs were equally easy to discriminate. The disk-triangle pair was one of the pairs eliciting poorer discrimination performance [47]. In this same study, the image of a cross tended to be poorly discriminated, regardless of the form with which it was compared [47]. Likewise, in spontaneous spatial exploration tasks, crosses elicited different responses than other geometric shapes (disks, squares and triangles). In fact, the fish tended to avoid cross stimuli [48] (see also [51] for similar evidence in other species).

To sum up, while zebrafish seem to be able to discriminate shapes, their performance seems to depend on multiple factors, including the task (different appetitive conditioning tasks, object-recognition or place-recognition), the size and the shape of the stimuli. It is still unclear if and how these different factors interact with each other and whether the same learning constraints apply to different tasks. Zebrafish are being increasingly used as a neurobiological and genetic model of cognitive functions and diseases. It is thus important to provide a more complete picture of their cognitive abilities and constraints, starting with basic tasks such as shape discrimination [39]. Likewise, it is important to provide researchers with a wide array of reliable behavioural tasks, which can be employed in this species.

Our research group has perfected a visual discrimination task, based on multiple forms of reward, that is quite effective in other fish species (e.g., [19, 52–56]). This behavioral task is based on an exit learning procedure, initially developed to test spatial orientation in gold fish [57], and later adopted to test spatial and numerical abilities of zebrafish [40, 41, 58, 59]. In this visual discrimination task, male fish are confined in a central tank with two potential exits leading to a rewarded external environment, where food, shelter and female conspecifics can be found. One of the exits is blocked and cannot be used by the animals. Each exit is marked by a different stimulus, providing the only cue for the animal to identify and pick the ‘correct’ exit. The first exit chosen by each fish and the total number of choices for each exit are measured in this task. This procedure has never been used to test visual discrimination in zebrafish so far.

One of the aims of the current study was thus to test whether this task could be adapted to zebrafish. Doing so, we also wanted to verify whether, also in this task, the performance of zebrafish will be influenced by the shapes to be discriminated (and in particular by the use of crosses as a stimulus). Two different shape pairs were thus used for the visual discrimination training, to verify whether the presence of a cross shape would affect animals’ performance in this task. Moreover, to better characterise zebrafish performance, two versions of this task were tested, using two different experimental apparatuses. The first apparatus (used in Exp. 1 and 3) was similar to that used in previous visual discrimination studies with other fish species [19, 52–56]. The second test arena (used in Exp. 2 and 4) was an original apparatus of different shape and size, never used before.

As mentioned above, the other main aim of this paper was to investigate amodal completion of partially occluded objects [11] in zebrafish. Despite the importance of zebrafish as a model of the vertebrate visual system, this fundamental ability has not yet been tested in this species. In the current study we thus aimed to fill this gap.

## Materials and methods

### Ethics statements

This research was carried out in the Animal Cognition and Neuroscience Laboratory (A.C.N. Lab.) of CIMeC (Center for Mind/Brain Sciences) at the University of Trento (Italy). All husbandry and experimental procedures complied with European Legislation for the Protection of Animals used for Scientific Purposes (Directive 2010/63/EU) and were previously authorized by the University of Trento’s Ethic Committee for the Experiments on Living Organisms and by the Italian Ministry of Health (auth. no. 1111-2015-PR and auth. no. 848/2020–PR issued pursuant to art. 31 of Legislative Decree 26/2014).

### Subjects and housing

The zebrafish (*Danio rerio*, wild type) used for all the experiments were taken from the breeding stocks of our laboratory (CIMeC, University of Trento). Thirty-six adult males (body length between 4 and 5 cm) were used as experimental subjects, while two females (not observed in experiments) were used as social reinforcements. Naïve subjects were used for each experiment. Specifically, nine subjects were used for Exp. 1, eight for Exp. 2, eight for Exp. 3 and eleven for Exp. 4.

All fish were fed with dry food, maintained under a 10:14-h light-dark cycle. During the training period, food was provided only as a reinforcement in the training apparatus. During the experimental phase, fish were maintained in glass-made home tanks (25 L capacity, 26° C) enriched with gravel and plants. The home tanks were divided in different sections (each hosting one male) by plastic walls presenting a plastic mesh window. Thus, visual and olfactory/chemical contact was maintained, while direct physical interaction was prevented. Contact with females was not provided in the rearing environment.

### Apparatuses

**Fig 1:**
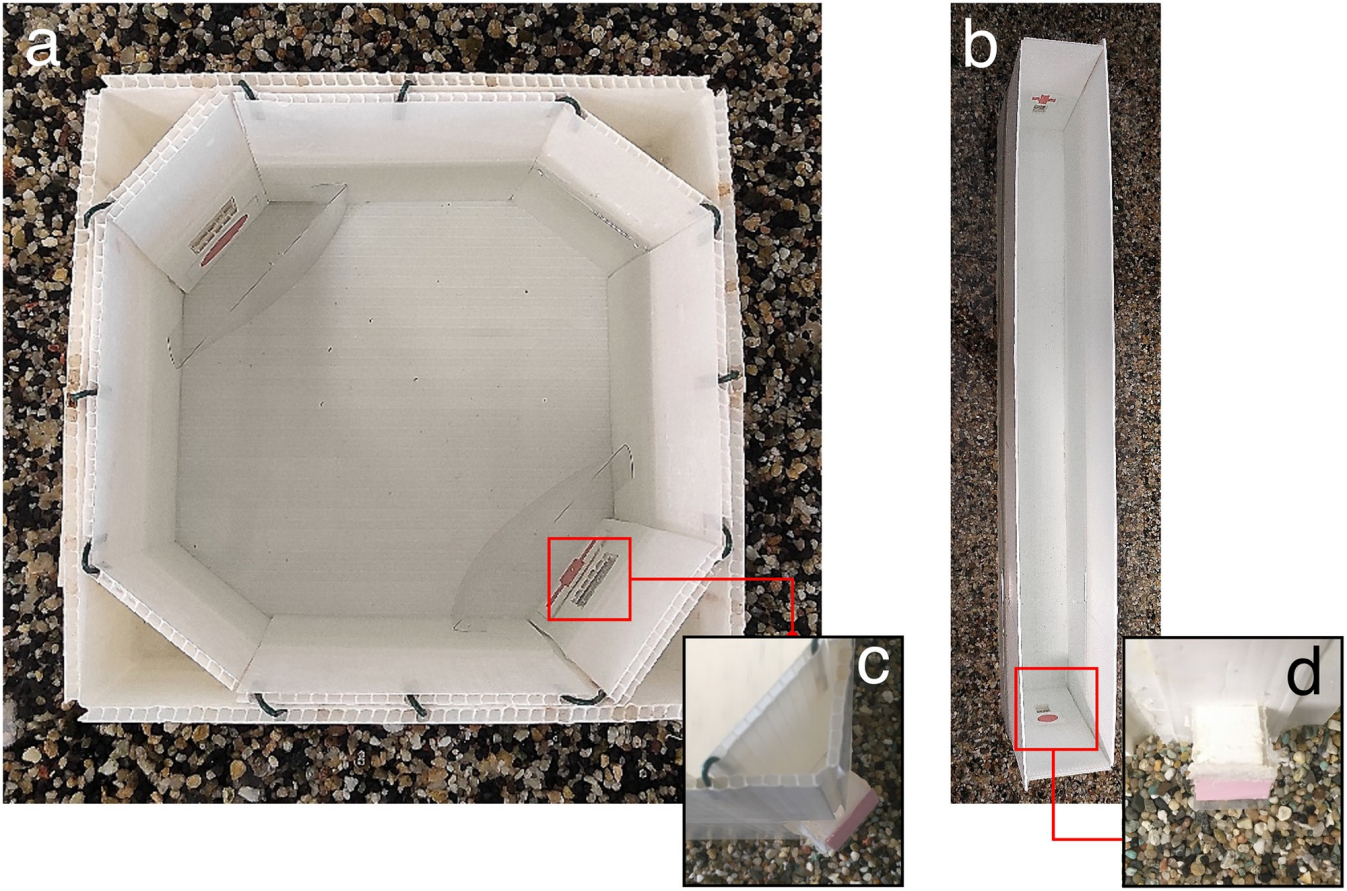
The square apparatus used in Exp. 1 and 3 (a) and the longitudinal apparatus used in Exp. 2 and 4 (b). For each apparatus, close up pictures of the tunnels are shown in (c) and (d).

Two different apparatuses were used for different experiments, in order to evaluate whether the use of different set-ups could influence zebrafish performance. The first apparatus consisted of a small square test tank, corresponding to that used in previous studies with other fish species [19, 52–56]. The second test apparatus was a long corridor (I-maze). In this set-up, more effort was required to express a choice, since fish had to swim longer to reach the reward, see below. This second apparatus was never used before and built *ad hoc* for the purpose of the present study. The small square apparatus, used in Exp. 1 and 3, consisted of a square test tank (15 x 15 x 15 cm) with uniform white plastic walls (Fig 1a). This test arena was inserted into a larger opaque rectangular tank (60 cm length x 36 cm width x 25 cm height). This created an external region, surrounding the square test arena. The external region was enriched with gravel, vegetation, food and two female conspecifics, as a reward. At two corners of the apparatus, on the same diagonal, a small tunnel (2.5 x 2 x 3 cm, placed 4.5 cm from the bottom of the tank) could lead to the surrounding rewarded region of the tank (Fig 1c). At the end of each tunnel there was a door (2.5 x 3.5 cm). The upper portion of each door (2.5 cm) was made of opaque plastic, while the lower portion (1 cm) was made of a transparent flexible plastic. This way, once a fish was inside one of the tunnels, it could see the environment on the other side of the door.

The two doors at the ends of the two tunnels were visually identical, but only one could be opened by the fish by pressing on it with the snout. The other door was blocked from the outside through a small wire hook. In this apparatus, visual stimuli (made of special plastic cardboard designed to withstand water) were placed below the entrance of each tunnel. Transparent screens (9 x 4 cm) were located 2.3 cm in front of the stimuli, so that the fish saw the stimuli from that minimum distance. The water level was kept constant, slightly above the corner exits and about 2 cm from the upper edge of the apparatus.

The second apparatus, used in Exp. 2 and 4, was a I-maze (Fig 1b). It consisted of a long corridor (70 x 8 x 20 cm), with uniform white plastic walls. At both ends of the apparatus, a small tunnel (2.5 x 2 x 3 cm; 4.5 cm from the bottom of the tank) was present. In this apparatus too, the tunnels lead to the external surrounding region, in which the fish could find the rewards. The rewarded region was created by inserting the I-maze into a larger opaque rectangular tank (110 x 45 x 52 cm), where vegetation, food and the two female conspecifics were present. As in the square apparatus, only one of the two doors could be opened by the fish. In this apparatus, the experimental stimuli were placed above each tunnel entrance. The water level (10 cm deep) was just above the upper edge of the two stimuli. This apparatus was designed to discourage repeated wrong choices, since its length greatly increased the effort required to reach each of the doors.

The apparatuses were placed in a darkened room and illuminated from above by either a 75 W light-bulb lamp (square apparatus) or an 18 W neon lamp (I-maze).

Above each of the apparatuses, a webcam was mounted to record the fish behaviour.

### Stimuli

**Fig 2:**
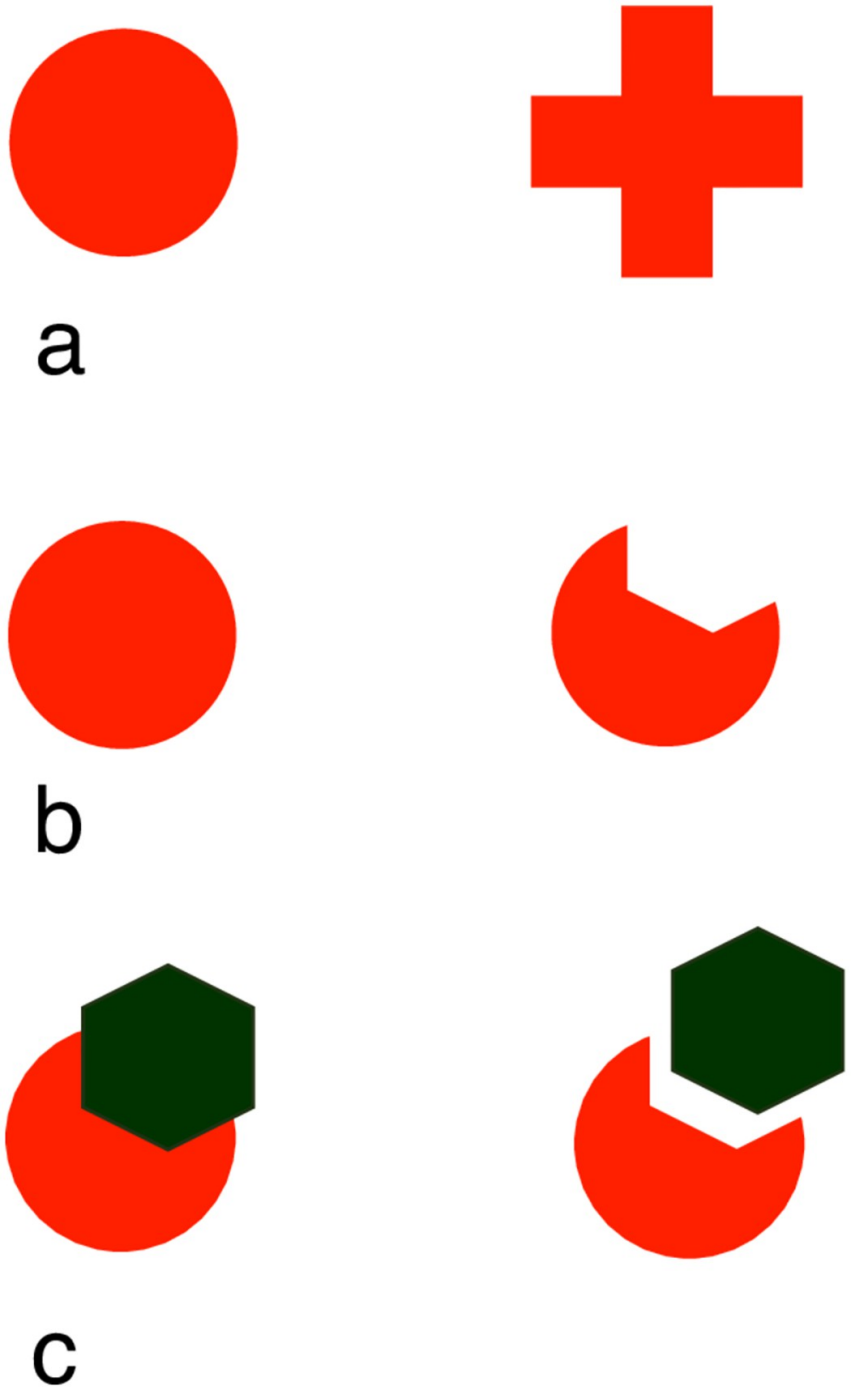
The training stimuli used in Exp. 1–2 (a) and Exp. 3–4 (b), as well as the test stimuli used for the amodal completion test in Exp. 3 and 4 (c). In this last pair of stimuli, the image on the left induces (in humans and other species) the perception of a full disk, amodally completing behind the dark green occluder, while the image on the right is perceived as an amputated disk placed close to the dark green occluder.

The stimuli used for Exp. 1 and 2 were two red figures (RGB: 255 red, 32 green, 0 blue): a disk (area 4.52 cm^2^; perimeter 7.54 cm) and a cross (area 4.52 cm^2^; perimeter 11.4 cm) (Fig 2a).

In Exp. 3 and 4, different stimuli were used. The training stimuli consisted of a full red disk (RGB: 255 red, 32 green, 0 blue; area 4.52 cm^2^; perimeter 7.54 cm) and an amputated red disk, which was missing a chunk of its upper portion (area 3.22 cm^2^; perimeter 7.74 cm) (Fig 2b). The stimuli used for the amodal completion test were similar to the training stimuli, with the addition of a dark green hexagonal polygon used as an occluder (RGB: 0 red, 51 green, 0 blue; width 2 cm; height 1.8 cm; side length 1 cm) (Fig 2c). In the two test stimuli, the occluder was either perfectly juxtaposed or just placed close to the amputated disk. To human observers, these configurations give the impression of representing respectively a full disk, partially hidden behind the green occluder (amodally completing behind it), or an amputated disk placed on the side of the occluder.

### Procedure

**Fig 3:**
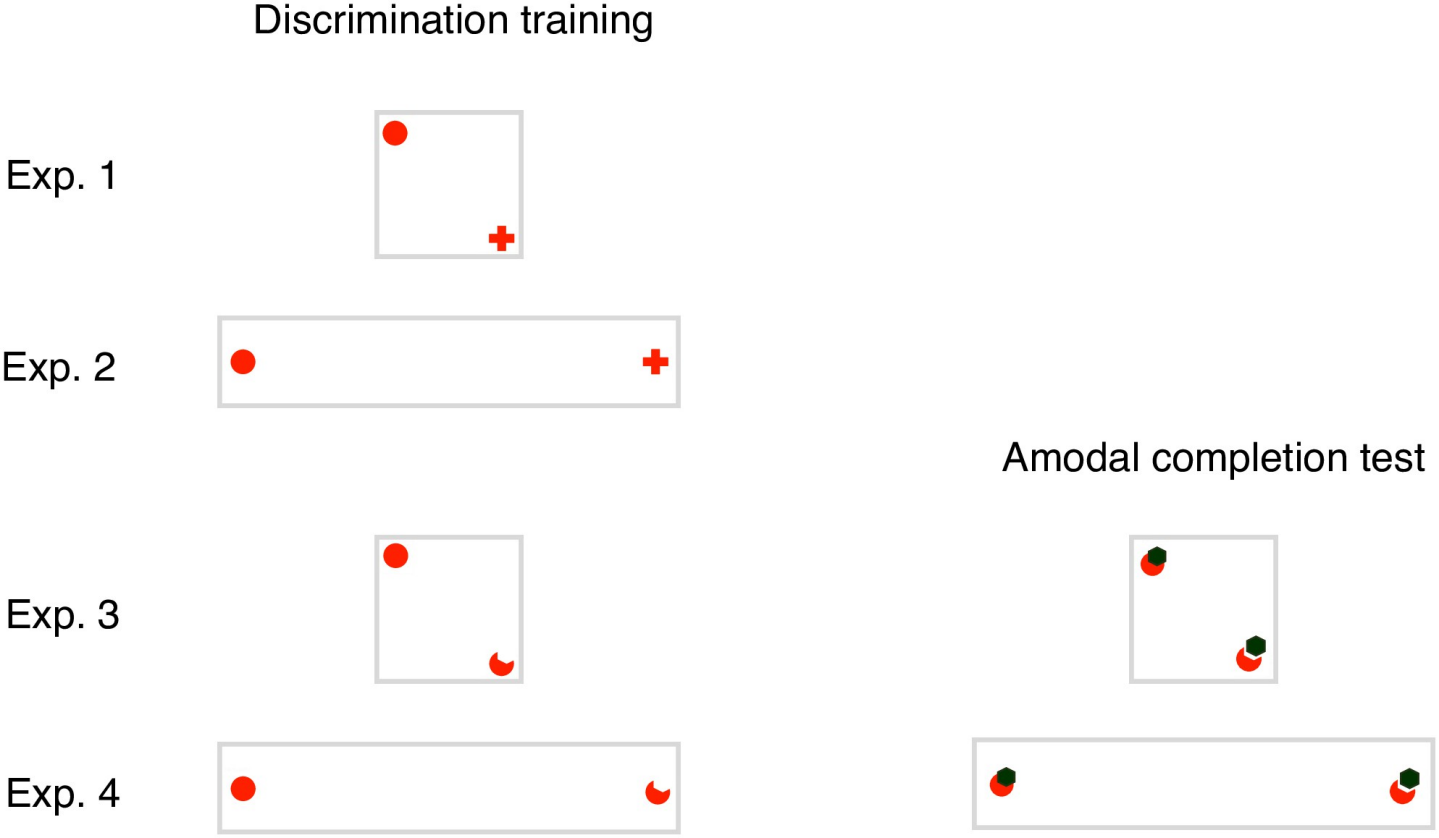
Overview of the experimental phases, apparatuses and stimuli used in the four experiments. Exp. 1 and 2 consisted only of a discrimination training, during which fish were trained to discriminate a disk and a cross. In Exp. 3 and 4, during the discrimination training, fish learned to discriminate between a full disk and an amputated disk. In these two experiments, after successfully reaching the learning criterion, the fish also participated to an amodal completion test. In Exp. 1 and 3 a small square apparatus was used, while in Exp. 2 and 4 an I-maze was used.

The general procedure consisted of two main experimental phases: a discrimination training, in which animals were trained to discriminate between two different visual stimuli, and an amodal completion test, in which animals were observed for their preference between a figure that elicited amodal completion perception and another that did not do so. The discrimination training was conducted in all four experiments (Exp. 1- Exp. 4). Moreover, in Exp. 3 and Exp. 4 only, after successfully completing the training, the animals underwent the amodal completion test. See Fig 3 for a schematic depiction of the design of the different experiments.

#### Discrimination training

In each experiment, fish were trained to discriminate between two visual stimuli (a red cross and a red disk in Exp. 1 and 3, Fig 2a; a full and an amputated red disk in Exp. 2 and 4, Fig 2b). In Exp. 1, five of the fish were reinforced for choosing the disk, while four for choosing the cross. In Exp. 2, four fish were reinforced on the disk and the remaining four on the cross. In Exp. 3, four fish were reinforced on the full disk and four on the amputated disk. In Exp. 4, six fish were reinforced on the full disk and five on the amputated disk.

In this task, fish had to choose the tunnel that was associated with the rewarded stimulus (which was counterbalanced among subjects, so that roughly the same number of animals was reinforced on each stimulus, see above). Reinforcement was provided by access to the rewarded region surrounding the test arena, where food and two female conspecifics were present (this has been shown to provide optimal motivation for this species, [60]).

At the beginning of each trial the fish was brought into the apparatus and delicately placed into a transparent plastic cylinder (diameter: 6 cm) in the centre of the apparatus. After 30 seconds, the cylinder was removed releasing the animal. A choice was scored whenever the animal entered one of the two tunnels (to score the choice the whole body had to be inside the tunnel). In similar exit learning tasks, zebrafish spontaneously tend to enter the tunnels, possibly reflecting a preference for small enclosed spaces over the open space of the test tank [40, 41, 58, 59] (see also [61], which exploits zebrafish tendency to spontaneously pass through small holes). Once inside the tunnels, they could see the external environment through the lower part of the door (made of transparent plastic), which motivated them to press it with their snouts and open it.

In each trial, the following criterion was used to determine whether the fish should receive the reward or not.

A trial was rewarded, if the fish visited the correct tunnel only and successfully got out from the test arena, without ever visiting the wrong tunnel. In this case, as a reinforcement, the fish was allowed to spend an inter-trial interval of 6 minutes in the external reward region, with females and food available.

If the fish visited the wrong tunnel, even only once, no reinforcement was given. However, to promote learning, a correction method was used in this case. After emitting a wrong choice, the fish was still allowed to perform other choices (i.e., to try to leave the apparatus entering the tunnels). The trial continued until either the fish was able to choose the right door and leave the arena, or until the time limit for the trial (10 minutes) was elapsed. In both cases, after the end of the trial the fish was allowed only 2 minutes in the external region, in the absence of food and females.

Note that, with this procedure, in each trial the fish could emit multiple choices for the wrong tunnel. Likewise, multiple choices could occur also for the correct tunnel. In fact, the fish could explore the correct door multiple times before actually exiting to the surrounding rewarded region and ending the trial.

If the fish failed to express any choice within the available 10 minutes, the trial was considered null. In this case, the fish would be placed in the external region and given a 5-minute inter-trial interval (without food and females). If 3 null trials were performed during the same day, the fish was placed back in its home tank until the next day. If the fish did not resume responding for 3 consecutive days, the training was interrupted and considered unsuccessful.

For Exp. 2 and 4, which used the I-maze, an additional shaping procedure was introduced. This was done to avoid that the animals would perseverate in their choices after choosing the closed door. This could happen due to the effort required to reach the other side of the apparatus, after emitting a choice. According to this correction procedure, after three consecutive choices for the non-rewarded door (within the same trial), the fish was confined to the other half of the apparatus.

In Exp. 1 and 3, the test arena was rotated 90° clockwise every two trials, to prevent the use of external cues. In Exp. 2 and 4, since it was not possible to rotate such a large apparatus, the position of the stimuli (and thus the blocked/openable door) was reversed during the training, following a semi-random sequence.

The dependent variables (behavioural measures) recorded during the training were the *first choice* performed in each trial and the *total number of choices* towards the two tunnels during each session. To test for inter-observer reliability, 10% of all trials were re-coded by a second coder, blind to the test condition of the fish (p<0.001 for Pearson’s correlation between the two coders, for all experiments).

Training was successfully completed when the fish reached a predefined learning criterion. The learning criterion required that the fish reached a 70% correct performance for both the dependent variables measured. This had to be achieved in each daily session, for 2 consecutive days. This means that, in each of the last two training sessions performed, the fish needed to reach both these criteria:

1. The fish had to emit at least 7 out of 10 correct first choices (i.e., in at least 7 out of 10 trials, the first tunnel entered by the fish was the correct one);
2. The total number of choices emitted for the correct tunnel (i.e., the total number of times the animal entered the correct tunnel) had to be at least 70% of the total number of choices emitted for either tunnel (i.e., the total number of times the animal entered either of the two tunnels).

Each animal could be trained for a maximum of 29 days. One additional training session could be performed if, on day 29, the performance was close to the learning criterion (e.g., if the learning criterion was fulfilled for one dependent variable only). Thus, fish received a maximum of 30 training sessions (each consisting of 10 trials), one per each day of the maximum allotted training period. Animals that did not reach the learning criterion within this period were considered unsuccessful learners and did not participate to further training.

#### Amodal completion test

In Exp. 3 and 4, after the animals were successfully trained to discriminate between a full and an amputated disk, they underwent an amodal completion test. The test began the day after the learning criterion was reached. The aim of the test was to verify whether the individuals reinforced on the full disk would choose the amodally-completing test stimulus, which to human observers appears as a full disk partially hidden behind the hexagonal occluder. On the contrary, the individuals reinforced on the amputated disk were expected to choose the test stimulus in which the gap between the red amputated disk and the green hexagon prevents the perception of amodal completion.

The test consisted of 10 trials (T), each lasting 2 minutes, followed by 3-minute intervals. During the test trials an extinction procedure was applied (i.e., both doors were closed), in order to avoid differential reinforcement of the two test stimuli. Test trials were intermixed with recall trials (R). During the recall trials, the training stimuli were present and the usual reinforcement procedures were carried out (to maintain the subjects’ motivation to the task as high as possible). The test session began with a pre-test set of two consecutive recall trials. If an incorrect response was scored in any of those two recall trials, further recall trials were administered until two consecutive correct responses were scored and the test could begin. During the test, test and recall trials were alternated according to the sequence T-R-T-R-T-R… If no mistakes were made during the recall trials, the test would consist of 10 test and 10 recall trials (split in two sessions, performed over 2 consecutive days). However, if a mistake was made in any of the recall trials, further recall trials had to be administered until two consecutive correct responses were scored and the test could progress. Thus, the number of recall trials performed during the test session could vary among the subjects (the number of test trials was always 10 for each individual). If too many additional recall trials were required, the test could be split over 3 days, to avoid fatiguing the animals.

### Statistical analysis

For each training session, the number of first choices for either stimulus was computed over the 10 trials (in each trial only one first choice could be emitted). A *first choice preference index* was then computed according to the formula:

$$\text{First}\;\text{choices}\;\text{for}\;\text{the}\;\text{disk}\;/\;\left(\text{first}\;\text{choices}\;\text{for}\;\text{the}\;\text{disk}+\text{first}\;\text{choices}\;\text{for}\;\text{the}\;\text{non-disk}\right)$$

The value of this index could range from 0 (indicating that all the first choices had been done for the non-disk) to 1 (all first choices for the disk), while 0.5 indicated an equal frequency of choices for the two stimuli.

Likewise, the overall number of total choices emitted for the two stimuli in the different trials was summed over the 10 trials of each session. This data was used to compute a *total choice preference index*, according to the same formula described above.

During the amodal completion test, the same dependent variables (computed according to equivalent formulas) reflected the number of first and total choices for the full disk. In this case, a value of 0 indicated exclusive choice for the amputated disk, while a value of 1 indicated exclusive choice for the full disk.

During the training phase, the values of these two indexes were used to evaluate whether each fish achieved the learning criterion (see above). For each fish, we also recorded the number of training trials needed to reach the learning criterion.

These two dependent variables (*first choice preference index* and *total choice preference index*) were also used to compare the training and test performance between the fish reinforced on the two different stimuli, within each experiment. In fact, in each experiment, significant learning will cause a significantly different performance in the animals reinforced on the (full) disk compared to the animals reinforced on the cross (or on the amputated disk, for Exp. 3 and 4). More specifically, the proportion of choices for the (full) disk should be higher in the former reinforcement group than in the latter reinforcement group. Likewise, during the amodal completion test, significant amodal completion perception can be deduced from a significant difference in the performance of the animals reinforced on the full disk and the animals reinforced on the amputated disk. In fact, the first group is expected to show a significantly higher proportion of choices for the stimulus eliciting amodal completion, compared to the second group.

In order to directly compare the performance of different groups of animals, two additional indexes were computed. These two indexes represented the preference for the rewarded training stimulus S+ (or for the test stimulus more corresponding to it). These indexes, named *first choice correct responses index* and *total choices correct responses index*, were calculated according to similar formulas as the previous ones, but in relation to the choices for the S+ stimulus. For instance, for a fish trained on the full disk vs. amputated disk discrimination and rewarded on the full disk, these two indexes will represent the proportion of first and total choices *for the full disk* (S+). However, for a fish reinforced on the amputated disk, the two indexes will represent the proportion of first and total choices *for the amputated disk* (S+).

Moreover, in order to study accuracy in the earlier vs. the later learning stages, fish performance was separately reported for the first and the second day in which the learning criterion was achieved (in fact the learning criterion required fish to reach a predefined performance: a minimum 70% correct responses for both dependent variables, in each of two consecutive daily sessions).

## Experiment 1: Disk vs. cross discrimination in the small square apparatus

The aim of this experiment was to verify if adult zebrafish can be trained to discriminate between two visual configurations, using a square apparatus (Fig 1a and 1c) and a procedure similar to those already validated in other fish species [19, 52–56]. The fish were trained to discriminate the disk and the cross stimuli, until the learning criterion was reached (Fig 2a).

### Results

Only two animals, one reinforced on the cross (fish 1) and the other on the disk (fish 4), were able to reach the learning criterion within the allotted time. See Table 1 for the performance of the two successful individuals. Two animals of this sample stopped responding before the end of the experiment, highlighting the difficulty of this task for zebrafish. See (S1 Data) for data on animals that did not complete the training.

**Table 1:** Final performance (expressed as proportion of first and total choices for the disk) of the two successful fish achieving the learning criterion in Exp. 1. For each animal the number of trials and sessions needed to reach the criterion (TTC—Trials To Criterion) is reported, as well as the performance level in the last two days of training, for both dependent variables.

	TTC	first choice index	total choice index	first choice index	total choice index
		(Day 1)	(Day 1)	(Day 2)	(Day 2)
Fish 1	142	1.0	1.0	0.7	0.71
(S+ disk)	(14 sessions)				
Fish 4	84	0.25	0.29	0.3	0.29
(S+ cross)	(8 sessions)				

## Experiment 2: Disk vs. cross discrimination in the long apparatus

The discrimination task tested in the first experiment was particularly challenging for the fish, most of which failed to reach the learning criterion. In the current experiment, as in Exp. 1, the fish were trained to discriminate the cross and the disk stimuli (Fig 2a). However, in this experiment, fish were trained within an I-maze (a long corridor with the two stimuli placed at the two ends, Fig 1b and 1d). The aim of this second experiment was thus to test whether the animals’ performance could be facilitated by the use of a different test apparatus, developed for discouraging repeated wrong choices.

### Results

As in Exp. 1, only two fish reached the learning criterion (or came reasonably close to it): fish 3 (reinforced on the disk) successfully reached the learning criterion, while the performance of fish 4 (reinforced on the cross) plateaued at about 67% correct choices and remained stable at this level for 3 days (see Table 2). One individual (fish 2, reinforced on the cross) stopped responding before completing the training period (see S1 Data for data on animals that did not complete the training).

**Table 2:** Final performance (as proportion of choices for the disk) of the two fish achieving or approaching the learning criterion in Exp. 2. For each animal the number of trials and sessions needed to reach the criterion (TTC—Trials To Criterion) is reported, as well as the performance level in the last two days of training, for both dependent variables.

	TTC	first choice index	total choice index	first choice index	total choice index	first choice index	total choice index
		(Day 1)	(Day 1)	(Day 2)	(Day 2)	(Day 3)	(Day 3)
Fish 3	30	0.90	0.91	0.90	0.90		
(S+ disk)	(3 sessions)						
Fish 4	173	0.33	0.33	0.33	0.35	0.25	0.33
(S+ cross)	(17 sessions)						

## Experiment 3: Full vs. amputated disk discrimination and amodal completion test in the small square apparatus

The first two experiments showed that the visual discrimination between images of a cross and a disk is particularly challenging for zebrafish. The aim of the current experiment was first to test whether a different visual discrimination (full vs. amputated disk, Fig 2b), would be easier for the animals. The same apparatus and procedure as in the first experiment were used, to ensure the comparability of the results. Moreover, if the animals could be successfully trained in this discrimination, the second aim of the experiment was to use this opportunity to test zebrafish susceptibility to amodal completion. Similar to what has been done with other fish species [19], this was done by testing their preference between the stimuli presented in Fig 2c. In these stimuli, an occluder was either perfectly juxtaposed or just placed close to the amputated disk (only the first configuration causes amodal completion of the disk in human observers).

### Results

#### Discrimination learning

**Fig 4:**
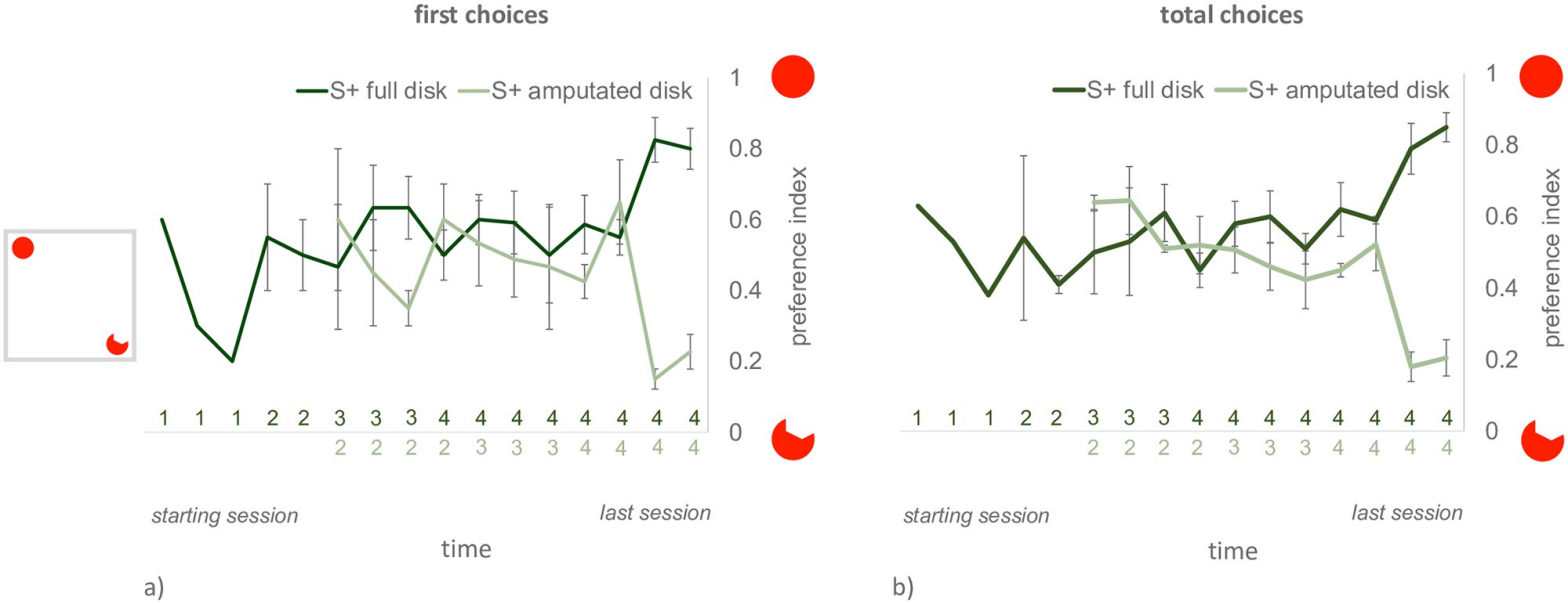
Learning curves for Exp. 3. The curve for the dependent variable first choice preference index is plotted on the left (a), while the curve for the total choices preference index is plotted on the right (b). On the y-axis, the level of preference for the full disk (higher values correspond to higher proportion of choices for the full disk). Darker lines represent the performance of the animals that at training were reinforced on the full disk, while the lighter lines represent the performance of animals reinforced on the amputated disk. Mean ± SEM are shown, for each training session. The learning curves of individual fish were anchored to their last training session, before being averaged. This was done to account for the different number of sessions each indfividual needed to reach the learning criterion. Each point represents the performance of a single training session (composed of 10 trials). Subsequent training sessions are ordered from left to right, in chronological order, from the beginning of the training until the achievement of the learning criterion. Data points for which no error bar is displayed represent sessions including one subject (since only one subject needed more than 13 training sessions to complete its training). The number of subjects participating to each training session is reported below the graph, close to the X-axis (the numbers of subjects reinforced on the full- and amputated-disk are reported, respectively, above and below the axis).

In this experiment, all the trained animals successfully reached the learning criterion (see Fig 4 for the learning curves). Fish reinforced on the full disk took on average 110.75 trials (SEM = 16.48, range 72–150) to reach the criterion, while individuals reinforced on the amputated disk took on average 72.75 trials (SEM = 17.15, range 30–101). No significant difference emerged between the two reinforcement groups in the number of trials needed to reach the criterion (t_6_ = 1.597, p = 0.161, 95% CI [-20.22, 96.22]).

**Fig 5:**
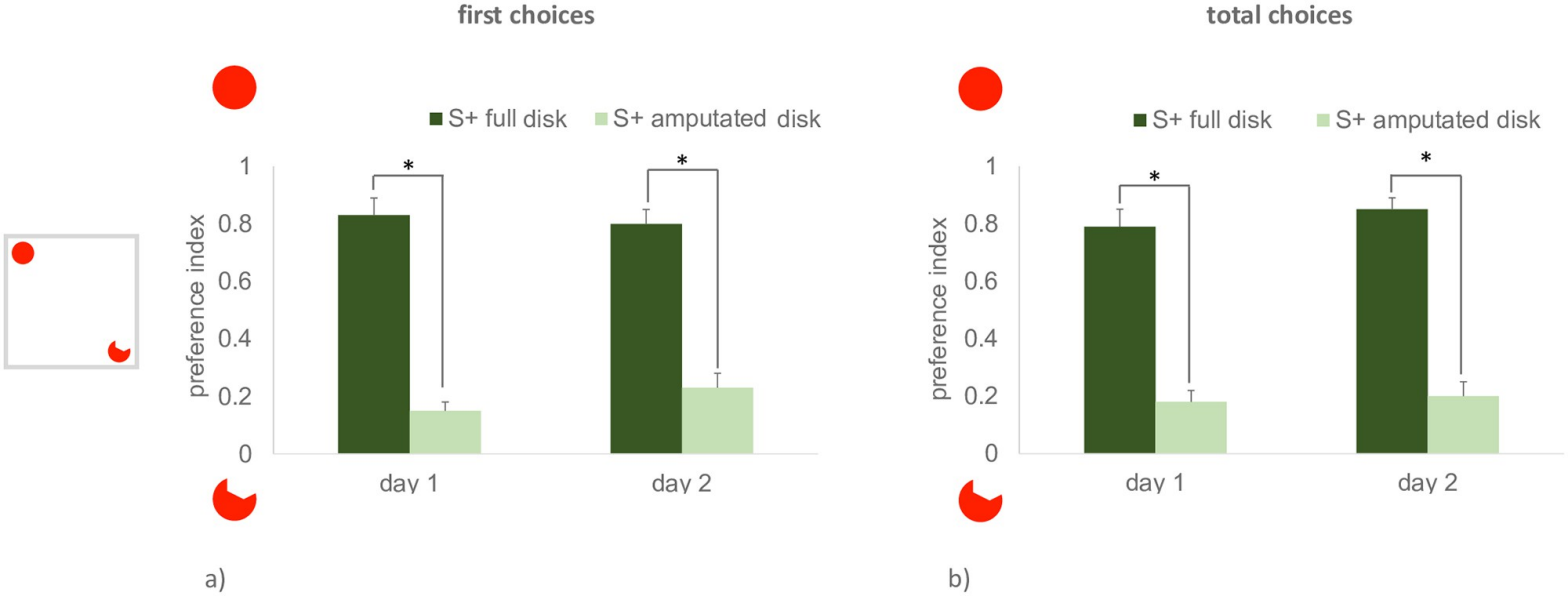
On the y-axis, the level of preference for the full disk displayed in the last two days of training in Exp. 3 (higher values correspond to higher proportion of choices for the full disk). The results for the two dependent variables, the first choice preference index and the total choices preference index are shown on the left and on the right, respectively. Darker and lighter columns are used to represent the performance of the animals reinforced on the full and on the amputated disk. Mean ± SEM is shown. Asterisks indicate significant departures from chance level. On the left side of the figure, a schematic view of the apparatus is depicted.

The level of preference for the disk in the last two training days was significantly different between the animals reinforced on the full vs. amputated disk, for both dependent variables, *first choice preference index* (day 1, (t_6_ = 9.751, p < 0.0001, 95% CI [0.506, 0.844]; day 2 t_6_ = 7.574, p < 0.001, 95% CI [0.388, 0.758], Fig 5a) and *total choices preference index* (day 1, t_6_ = 7.593, p < 0.001, 95% CI [0.415, 0.811]; day 2, t_6_ = 9.767, p < 0.001, 95% CI [0.482, 0.804], Fig 5b). This shows that the training procedure was successful in making fish performance diverge.

However, when the absolute performance of the two groups was considered, comparing the values of the *first choice correct responses index* and *total choices correct responses index*, no difference was found between the two groups (day 1, first choices t_6_ = -0.361, p = 0.730, 95% CI [-0.194, 0.144]; total choices t_6_ = -0.363, p = 0.729, 95% CI [-0.228, 0.169]; day 2, first choices t_6_ = 0.174, p = 0.868, 95% CI [-0.163, 0.188]; total choices, t_6_ = 0.680, p = 0.522, 95% CI [-0.100, 0.177]).

Since no difference emerged between the two reinforcement groups, data from the two groups was merged. In this merged sample, the absolute performance was above chance level (day 1, first choices t_7_ = 10.420, p < 0.001, 95% CI [0.260, 0.414]; total choices t_7_ = 8.030, p < 0.001, 95% CI [0.215, 0.395]; day 2, first choices t_7_ = 8.815, p < 0.001, 95% CI [0.215, 0.372]; total choices t_7_ = 12.051, p < 0.001, 95% CI [0.264, 0.394]). This indicates significant choice for the reinforced stimulus, at the end of the training.

#### Amodal completion test

**Fig 6:**
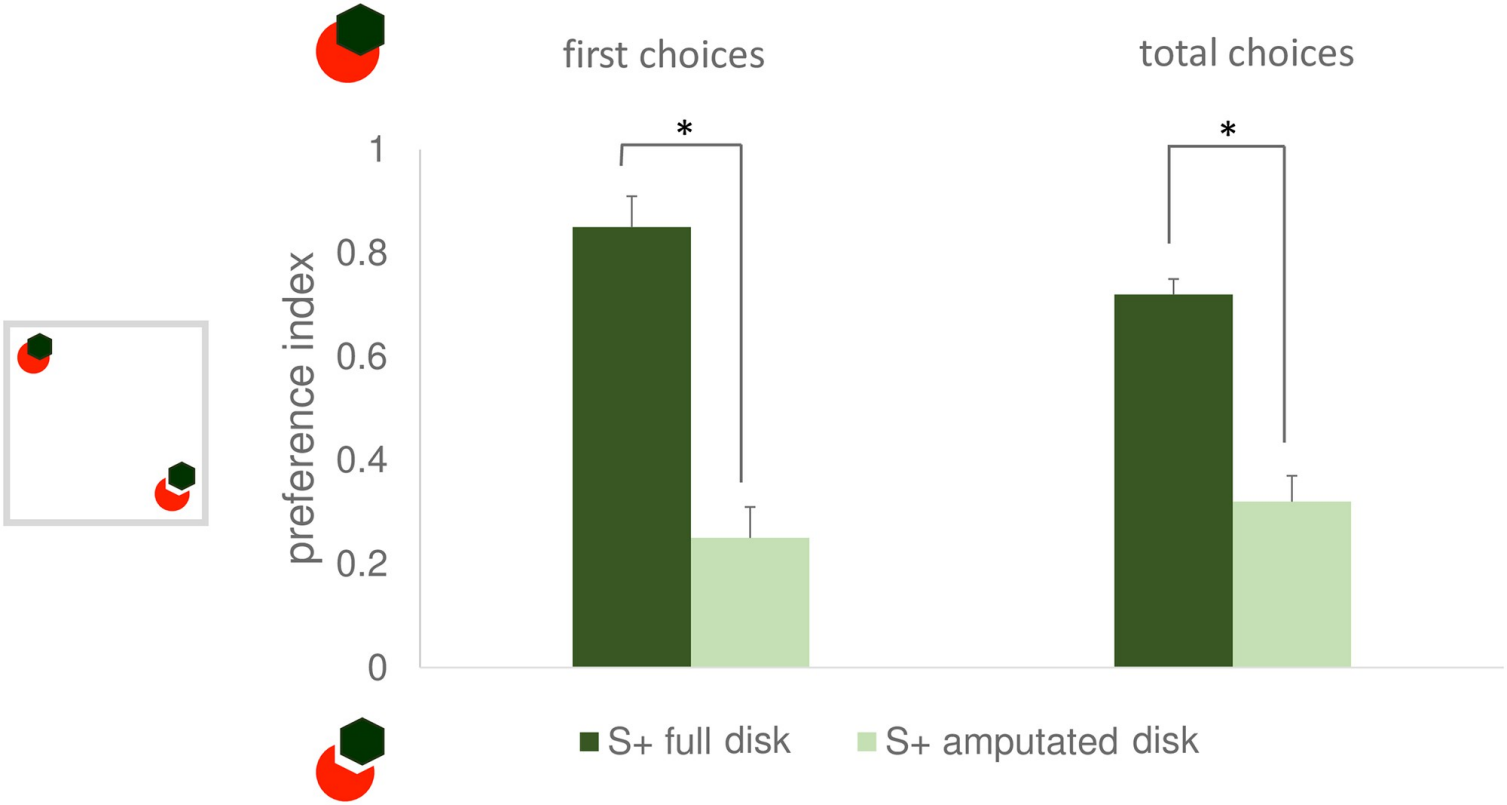
Level of preference for the amodally completing disk displayed during the amodal completion test of Exp. 3 (higher values correspond to higher proportion of choices for the amodally completing disk). The results for the two dependent variables, the first choice preference index and the total choices preference index are shown on the left and on the right, respectively. Darker and lighter columns are used to represent the performance of the animals reinforced on the full and on the amputated disk. Mean ± SEM is shown. Asterisks indicate significant departures from chance level. On the left, a schematic view of the apparatus is depicted.

In line with what seen for the discrimination learning, for the amodal completion test too, significant differences were found in the *first choice preference index* (t_6_ = 6.573, p = 0.001, 95% CI [0.377, 0.823]) and in the *total choices preference index* (t_6_ = 6.614, P = 0.001, 95% CI [0.253, 0.549]) (Fig 6). This is an evidence of amodal completion perception. In fact, compared to the fish reinforced on the amputated disk, the fish reinforced on the full disk showed higher proportion of choices for the stimulus that should elicit amodal completion of the disk.

No significant differences between the two groups were found in the corresponding indexes of absolute performance (*first choice correct responses index* t_6_ = 1.095, p = 0.315, 95% CI [-0.123, 0.323]; *total choices correct responses index* t_6_ = 0.670, p = 0.528, 95% CI [-0.106, 0.186]). When data of the two reinforcement groups was merged, absolute performance was clearly above chance (first choices t_7_ = 6.481, p <0.001, 95% CI [0.190, 0.409]; total choices t_7_ = 6.978, p < 0.001, 95% CI [0.132, 0.267]). This confirms the presence of a significant amodal completion effect. In fact, fish of both reinforcement groups chose the stimulus that is more similar to their reinforced training stimulus S+, if amodal completion is correctly perceived.

## Experiment 4: Full vs. amputated disk discrimination and amodal completion test in the long apparatus

The aim of the last experiment was to test whether the use of a different apparatus (the same I-maze of Exp. 2) would affect fish performance in the discrimination training or in the amodal completion test.

### Results

#### Discrimination learning

**Fig 7:**
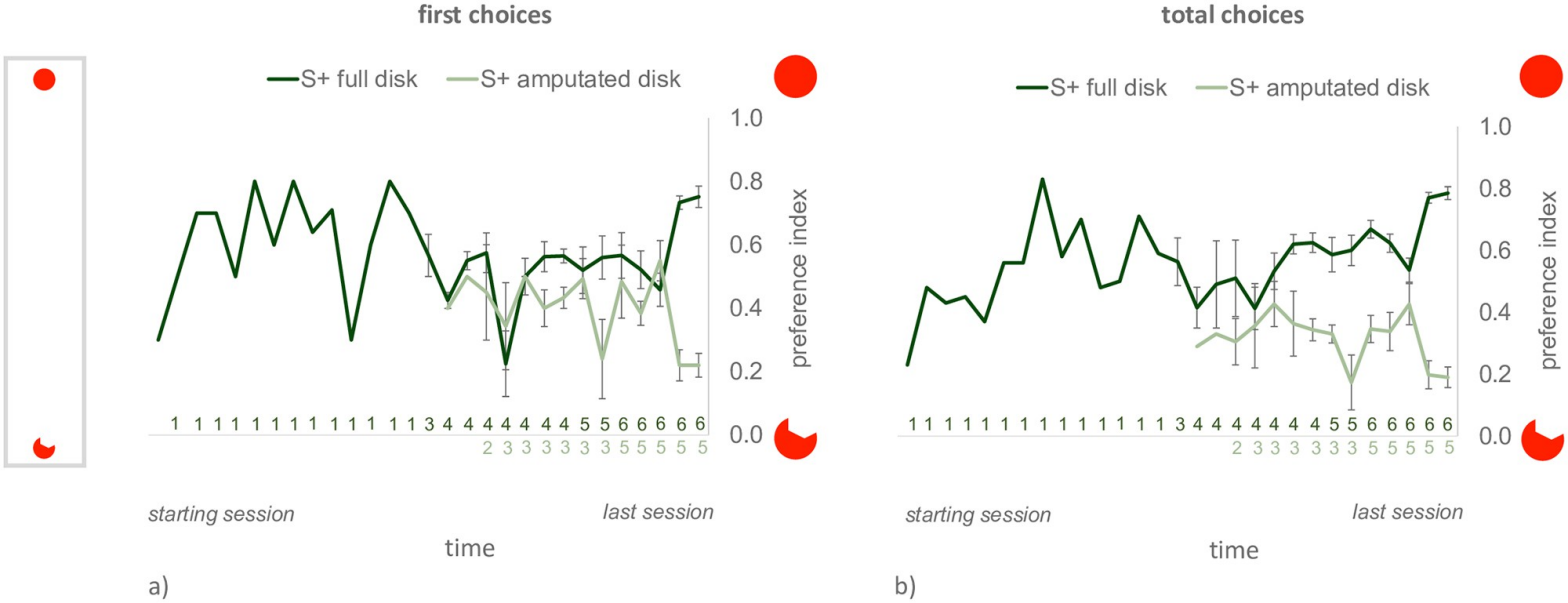
Learning curves for Exp. 4. The curves for the first choice preference index and for the total choices preference index are plotted on the left (a) and on the right (b) respectively. On the y-axis, the level of preference for the full disk. Darker and lighter lines represent the performance of the animals reinforced on the full or amputated disk, respectively. Mean ± SEM are shown, for each training session. The learning curves of individual fish were anchored to their last training session, before being averaged. Subsequent training sessions are ordered, chronologically, from left to right. Data points for which no error bar is displayed represent sessions with only one subject (which needed a higher number of training sessions to reach the criterion). The number of subjects participating to each training session is reported below the graph, close to the X-axis (the numbers of subjects reinforced on the full- and amputated-disk are reported, respectively, above and below the axis).

In this case also, all the animals reached the learning criterion (see Fig 7 for the learning curves). Fish reinforced on the full disk took on average 133.33 trials (SEM = 34.61, range 40–281) to reach the criterion, while individuals reinforced on the amputated disk took on average 85.4 trials (SEM = 19.08, range 40–132). No significant difference emerged between the two reinforcement groups in the number of trials needed to reach criterion (t_9_ = 1.142, p = 0.283, 95% CI [-47.00, 142.86]).

**Fig 8:**
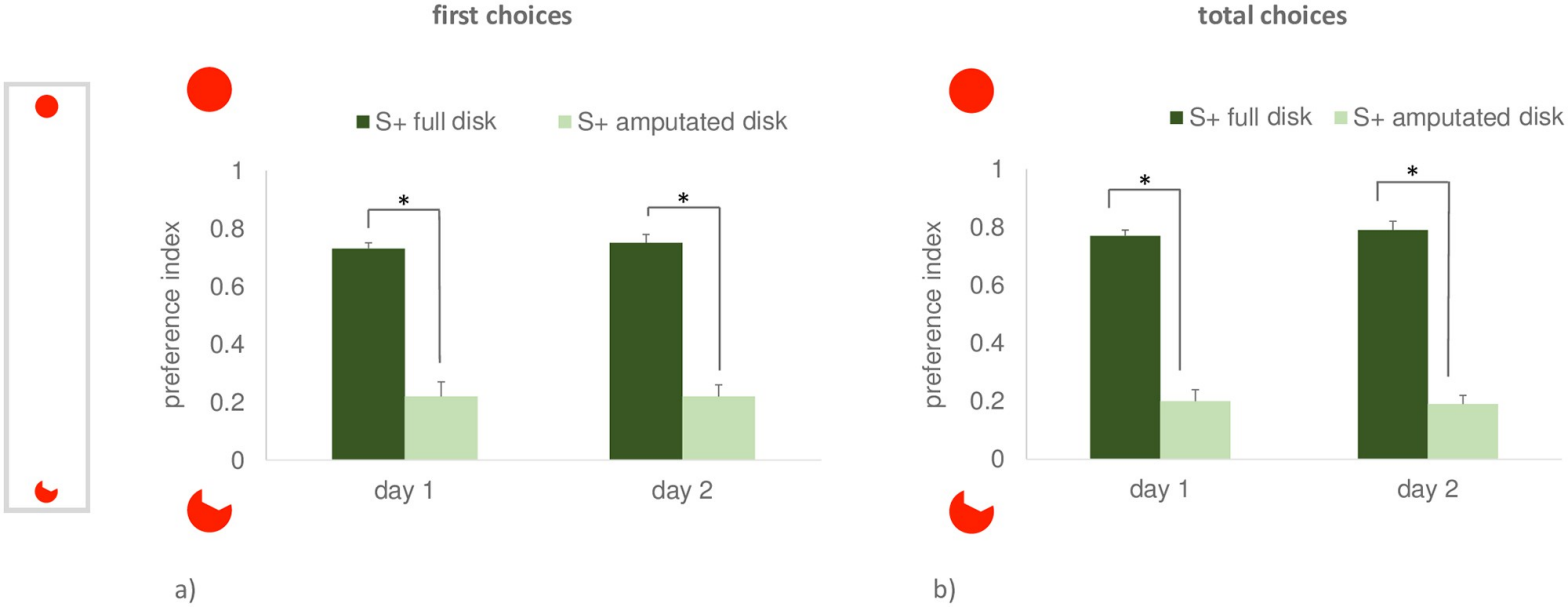
Results of the last two days of training in Exp. 4. The first choice preference index is shown on the left (a), while the total choices preference index is shown on the right (b). Darker and lighter columns represent the performance of the animals reinforced on the full and on the amputated disk, respectively. Mean ± SEM is shown. Asterisks indicate significant departures from chance level. On the left, a schematic view of the apparatus.

Once again, the level of preference for the full disk in the last two training days was significantly different between the animals reinforced on the full vs. amputated disk, for both dependent variables, *first choice preference index* (day 1 t_5.46_ = 5.469, p <0.001, 95% CI [0.38, 0.65], please note that violations of homoscedasticity were found by Levene’s test F = 19.180, p = 0.002, and appropriate corrections were applied; day 2 t_9_ = 10.615, p ≤ 0.0001, 95% CI [0.42, 0.65]), and *total choices preference index* (day 1, t_4.96_ = 12.218, p <0.001, 95% CI [0.45, 0.70], also here violations of homoscedasticity were found by Levene’s test, F = 12.439, p = 0.006, and corrected values are reported; day 2 t_9_ = 13.288, p ≤ 0.001, 95% CI [0.49, 0.7]) (see Fig 8a and 8b respectively for the two dependent variables). As in the previous experiment, this is what we expected after successful training.

When the absolute performance of the two groups was considered, comparing the values of the *first choice correct responses index* and *total choices correct responses index*, no difference was found between the two groups (day 1, first choices t_5.46_ = -0.875, p = 0.418, 95% CI [-0.18, 0.08], violations of homoscedasticity were detected F = 19.18, p = 0.002 and corrected values are reported; total choices t_4.92_ = -0.651, p = 0.544, 95% CI [-0.15, 0.09]; day 2, first choices t_9_ = -0.551, p = 0.595, 95% CI [-0.14, 0.08]; total choices t_9_ = -0.551, p = 0.614, 95% CI [-0.13, 0.08]). As in the previous experiment, this indicates that the final level of performance achieved at training is not affected by whether the reinforced stimulus (S+) was the full or the amputated disk. When data of the two reinforcement groups was merged, the absolute performance of the merged sample was above chance level (day 1, first choices t_10_ = 10.293, p<0.001, 95% CI [0.2, 0.30]; total choices t_10_ = 13.22, p<0.001, 95% CI [0.24, 0.33]; day 2 first choices t_10_ = 10.99, p<0.001, 95% CI [0.21, 0.32]; total choices t_10_ = 13.67, p<0.001, 95% CI [0.25, 0.35]. This indicates significant choice for the reinforced stimulus.

#### Amodal completion test

**Fig 9:**
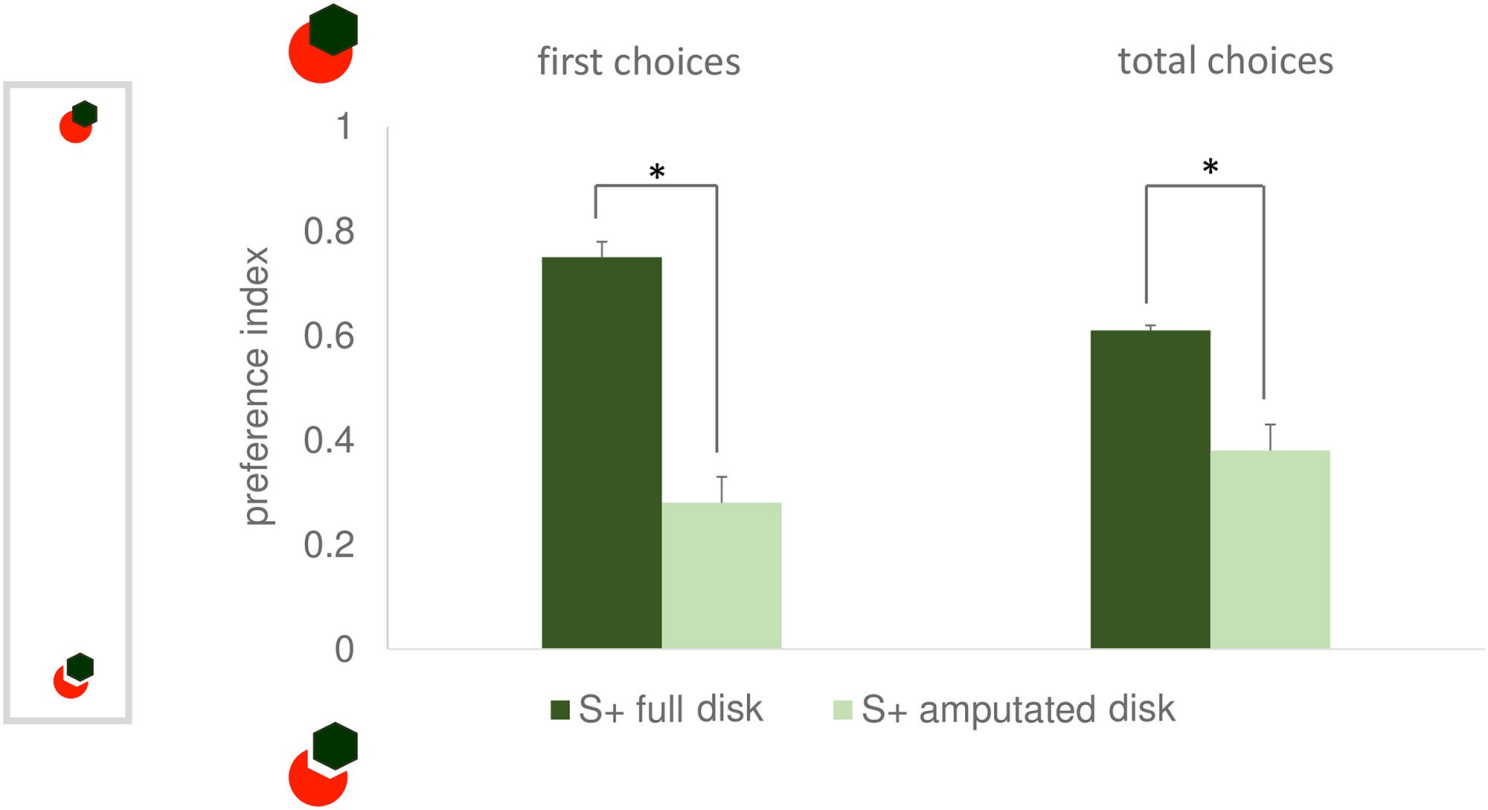
Results of the amodal completion test of Exp. 4. The first choice preference index and the total choices disk preference are shown on the left and on the right, respectively. Darker and lighter columns are used to represent the performance of the animals reinforced on the full and on the amputated disk. Mean ± SEM is shown. Asterisks indicate significant departures from chance level. On the left, a schematic view of the apparatus is depicted.

In the amodal completion test too, significant differences were found between the two reinforced groups in the *first choice preference index* (t_9_ = 8.082, p < 0.001, 95% CI [0.34, 0.60]) and in the *total choices preference index* (t_4.404_ = 4.525, p = 0.008, 95% CI [0.09, 0.36], violations of homoscedasticity were detected F = 9.212, p = 0.014 and corrected values are reported) (Fig 9). This is an evidence of amodal completion perception, since, compared to the fish reinforced on the amputated disk, the fish reinforced on the full disk showed higher proportion of choices for the stimulus that should elicit amodal completion perception.

On the contrary, no significant differences were detected between the two groups in the corresponding indexes of absolute performance (first choices: t_9_ = 0.516, p = 0.618, 95% CI [-0.101, 0.161]; total choices: t_4.45_ = -0.173, p = 0.870, 95% CI [-0.14, 0.12], violations of homoscedasticity were detected F = 8.429, p = 0.018 and corrected values are reported). When data of the two reinforcement groups was merged, absolute performance was clearly above chance (first choices t_10_ = 8.48, p < 0.001, 95% CI [0.17, 0.3]; tot choices t_10_ = 5.237, p < 0.001, 95% CI [0.07, 0.16]). This confirms the presence of a significant amodal completion effect. In fact, fish of both reinforcement groups chose the stimulus that is more similar to their reinforced training stimulus S+, if amodal completion is correctly perceived.

### Between-experiment comparisons

**Fig 10:**
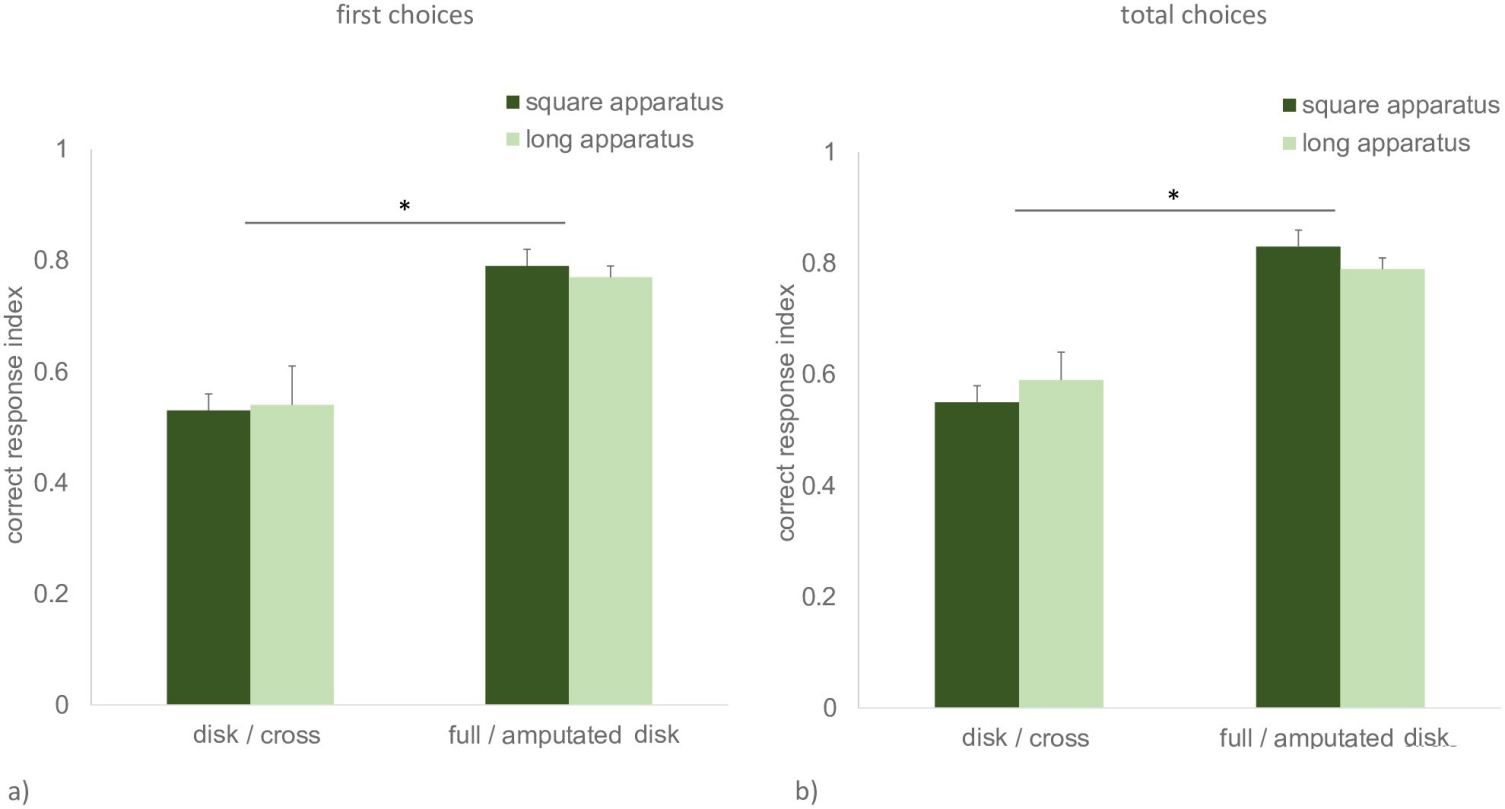
Between-experiment comparisons of the level of performance (i.e., proportion of choices for the reinforced stimulus) on the last two days of training. Graph (a) reports data for the first choice correct responses index, while graph (b) reports data for the total choices correct responses index. Darker columns represent the values for animals trained in the square apparatus (Exp. 1 and 3), while lighter columns indicate the values for animals trained in the long apparatus (Exp. 2 and 4). Within each graph, the two columns on the left represent the values for Exp. 1 and 2 (cross vs. disk discrimination), while the two columns on the right represent Exp. 3 and 4 (full vs. amputated disk discrimination). Mean ± SEM is shown. Asterisks indicate significant departures from chance level.

To test the effect of the apparatus and of the stimuli used, additional between-experiment comparisons were conducted. To this aim, a multivariate ANOVA was run on the *first choice correct responses index* and *total choices correct responses index*, studying the effects of two between-subject factors (*apparatus*, 2 levels: long and square; *stimuli*, 2 levels: disk/cross and full/amputated disk). A significant effect of stimuli was detected, for both dependent variables (first choices: F_(1,32)_ = 35.12, p < 0.001; total choices, F_(1,32)_ = 51.41, p < 0.001). On the contrary, no significant effect was detected for the factor *apparatus* (first choices, F_(1,32)_ = 0.09, p = 0.767; total choices, F_(1,32)_ < 0.001, p = 0.994), nor any significant interaction between the two factors (first choices F_(1,32)_ = 0.161, p = 0.691; total choices, F_(1,32)_ = 0.908, p = 0.348) (Fig 10).

**Fig 11:**
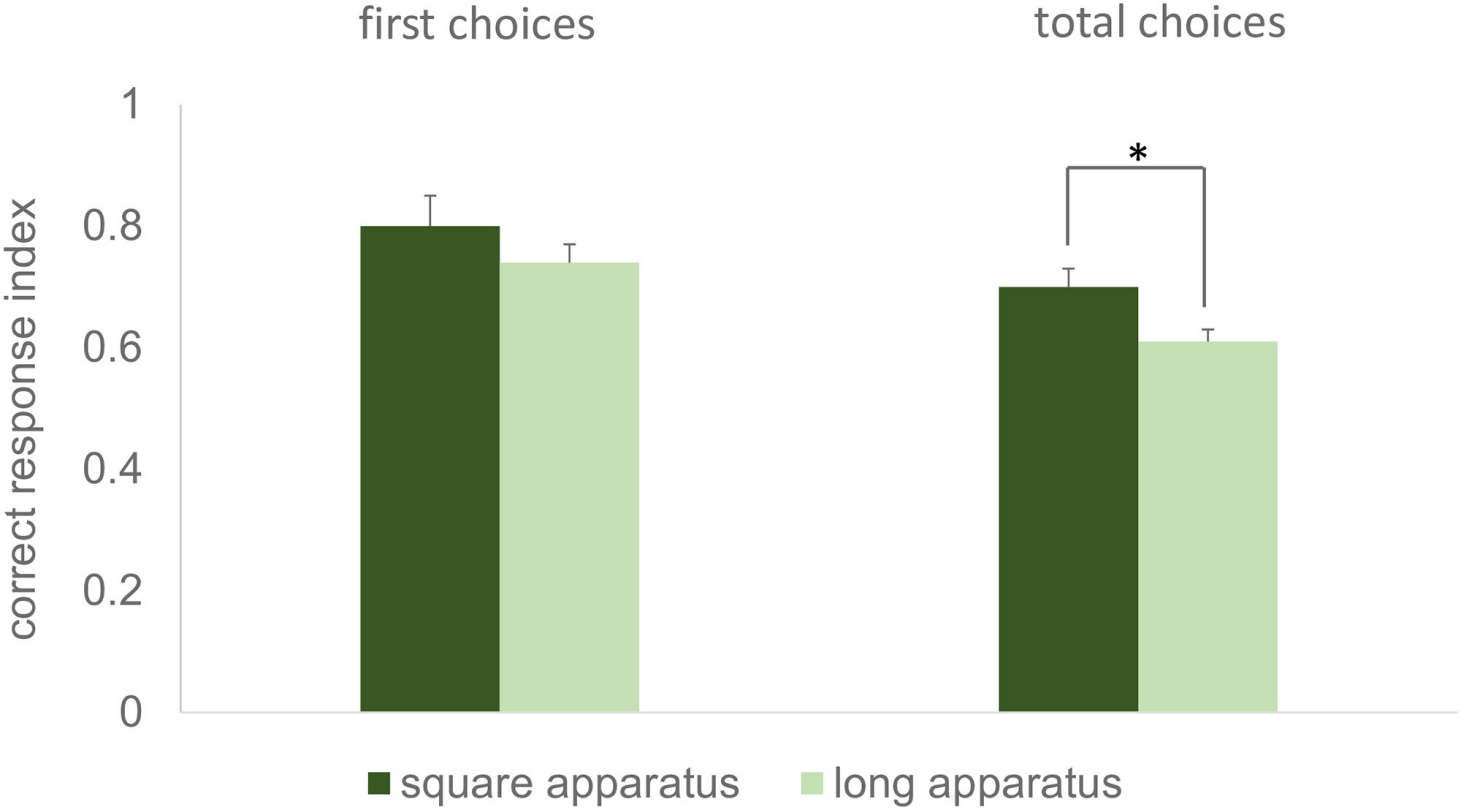
Comparison of the performance observed in the amodal completion test in Exp. 3 (training in the square apparatus, darker columns) and in Exp. 4 (training in the long apparatus, lighter columns). The results for the first choice correct responses index and for the total choices correct responses index are shown on the left and right side of the graph, respectively. Mean ± SEM is shown. Asterisks indicate significant departures from chance level.

Moreover, an independent sample t-test was conducted to compare the performance of the fish trained in the square and in the long apparatus, in the amodal completion test. No difference was found in the performance of the fish observed in Exp. 3 and 4, when the *first choice correct response index* was considered (t_17_ = 1.246, p = 0.230, 95% CI [-0.04, 0.17]). However, when the *total choices correct responses index* was considered, a significant difference emerged, with the group of fish trained in the square apparatus showing significantly higher performance (t_17_ = 2.465, p = 0.025, 95% CI [0.01, 0.16], Fig 11).

Since no significant difference emerged between Exp. 3 and 4 for the *first choice correct response index*, data of these two experiments were merged and the resulting sample was compared against chance level, by a one-sample t-test. This confirmed the results obtained for each experiment, showing that absolute performance was above chance also for this combined sample (t_18_ = 10.278, p < 0.001, 95% CI [0.21, 0.32]).

## General discussion

In the current study, we obtained two very important findings. On the one hand, we validated two versions of a visual discrimination learning task, showing that zebrafish can be successfully trained to discriminate between two visual shapes in order to reach a location where conspecifics, food and shelter can be found. On the other hand, we reported the first evidence of amodal completion perception in this species. We will now discuss these two main results, in this order.

As for the first main result, we now know that one of the tasks which have previously been used to investigate visual perception in another fish species (*Xenotoca eiseni*) [19, 52–56], can be applied to zebrafish too. In this task, the animals learn to locate the tunnel that leads to the external rewarded region, based on the stimulus presented close to its entrance. This allows them to receive a reward consisting of food and exposure to females in a comfortable environment. Zebrafish could successfully solve this task both when it was implemented in an apparatus similar to that used for previous studies [19, 52–56] and when it was implemented in a new I-maze. This paves the way for future studies comparing visual perception among different fish species, while also providing another tool for research based on visual discrimination training in this crucial animal model. This is particularly relevant in the light of the recent debate on the extent of zebrafish ability to perform shape discrimination (e.g., [39]).

However, not all kinds of visual discrimination were equally easy for zebrafish. The first two experiments revealed that, for zebrafish, the discrimination between a disk and a cross is particularly difficult to learn, with only two fish reaching or approaching the learning criterion in each experiment. In line with that, in these two experiments some subjects stopped responding before the end of the training. We are speculating that this could reflect a response to the difficulty of the task. The inability to solve the visual discrimination task seems, in fact, the most likely explanation for the fact that some animals stopped responding in the first two experiments. Factors such as over-habituation to the general procedures/set-ups and loss of general motivation to reach the rewards are unlikely to be the cause of this behaviour. Indeed, these aspects were identical to Exp. 3 and 4, in which no animal stopped responding before reaching the end of the training.

In contrast to the first two experiments, in the last two experiments, all the subjects trained to discriminate a full disk from an amputated disk were able to reach the learning criterion. Successful learning was also shown by the significant difference in the proportion of choices for the full disk that could be observed, at the end of the training, between the two reinforcement groups (animals reinforced on the full or amputated disk). In fact, at the end of the training, animals that had been reinforced on the full disk showed a higher proportion of choices for this stimulus, compared to animals that had been reinforced on the amputated disk. However, when we looked at the proportion of correct responses (i.e., the preference for the rewarded stimulus for each group), the performance of the two groups did not differ. This indicates that animals reinforced on the full and on the amputated disk had a similar learning performance, without any evidence of spontaneous preferences for any of the two stimuli. When data from the two training groups was merged, performance was significantly above chance, further demonstrating that we achieved significant learning of the trained discrimination in both these two groups.

The comparison between the first and the last two experiments shows that not all visual discriminations are equally easy to learn for this model species. This appears to be in line with previous studies reporting uneven performances in zebrafish trained to discriminate different shape pairs, and specifically with crosses ([47, 48] see the Introduction and see also [51, 62–66] for evidence in other fish species). One hypothesis thus could be that, also in the task employed here, zebrafish show uneven performance when different shape pairs are used.

This could be particularly relevant in the light of the current debate on the impact of different testing procedures on zebrafish shape discrimination performance (e.g., [39, 45, 50]). Intriguingly, it has been recently shown that, for zebrafish, shape discrimination is easier when outlined figures are used, compared to when filled figures are used [46]. While the mechanisms behind this effect are still unclear, future studies could investigate whether, in the current task too, fish performance for the cross/disk discrimination could be improved using outlined instead of filled figures. Moreover, previous literature indicates that zebrafish performance in the discrimination of different shapes may be size dependent [39] (but see [46] for evidence of successful discrimination of small shapes). Future studies could thus investigate if, also in the task we used here, zebrafish shape discrimination is impacted by the size of the stimuli. For instance, it could be interesting to test whether the performance in the cross vs. disk discrimination can be rescued by increasing the stimuli size.

Another interesting explanation for the different performance observed in Exp. 1–2 vs. 3–4 could be that zebrafish might find it easier to discriminate between local differences in similar shapes (full vs. amputated disk) rather than between two completely different shapes (disk vs. cross). This would imply a processing style that favours the local details of the image, over its global configuration. The presence of a preference for the global or local aspects of a visual image has been tested in five fish species, including zebrafish [52, 67]. To do so, fish were tested with composite hierarchical configurations like those used to test the Navon effect [68] in human subjects. This paradigm allows to study the perception of the global and local dimensions of visual stimuli. This way, researchers can reveal whether, in a given species or task, the animals prioritize the processing of the figure as a whole or of its local details. When observed with stimuli in which the local and global information are put in conflict, most fish species prioritised the global aspects of the configuration over its local details. Only zebrafish showed no clear preference between local and global information, revealing an ambiguous profile for this task. Based on this evidence, it is difficult to conclude whether zebrafish might indeed find it easier to discriminate between local differences in similar shapes. Future studies could thus be devoted to test, whether, under other test conditions (e.g., changing the size of the stimuli), zebrafish would show a preference for local information in this task, highlighting a potential mechanism underlying the different performance in the cross/disk and in the full/amputated disk discriminations.

Finally, a last explanation of the different results obtained in the first and the last two experiments, might be related to the differences in areas between the two stimuli of each pair. Zebrafish have been shown to be particularly accurate in item size discrimination tasks [61]. Thus, it is conceivable that it would be easier for them to discriminate objects characterised by different area, rather than by different shape. While in Exp. 1 and 2 stimuli were matched in area, this was not possible in Exp. 3 and 4: in fact, an amputated circle must necessarily have a smaller area than a full circle. As a consequence, in Exp. 3 and 4, the two stimuli were matched by perimeter rather than by area. It is therefore plausible to speculate that zebrafish might be more sensitive to differences in area than to differences in perimeter, making the stimuli of the last two experiments easier to discriminate than those of the first two experiments.

In the current study, we did not find any evidence that subjects’ learning performance was affected by identity of the rewarded stimulus (i.e., whether S+ was the full or the amputated disk for a given animal). Indeed, in the first two experiments, the subjects that reached or approached the learning criterion were equally distributed between the two reinforcement groups. Likewise, in the last two experiments, no differences emerged between the fish reinforced on the full or amputated disk, when any index of absolute performance was considered. Even though non-significant results should always be interpreted with caution, our results seem to suggest that the differences we detected between the first and the last two experiments originate from differential discriminability of the two pairs of stimuli, rather than from a pre-existing preference for one of the training stimuli used. This is in contrast with the results of Cognato et al. [48], which seemed to suggest that images of crosses elicited a selective avoidance.

The apparatus used to train the animals did not seem to be the main factor driving their performance either. Indeed, fish mostly failed to complete the training in Exp. 1 and 2, regardless of the apparatus used, while they were all successful in Exp. 3 and 4. However, at least one of the two dependent variables (total choices) allowed us to detect a difference in the amodal completion test performance, depending on the apparatus used. During the amodal completion test, fish observed in the small square apparatus showed a significantly higher proportion of total choices for the correct stimulus than fish observed in the longitudinal apparatus. This effect emerged only for the total choices, but not when the first choices were considered. Thus, for the fish observed in the small square apparatus, performance seems to be more stably guided by what they learned during the training. This result was unexpected, since the longitudinal apparatus was devised to improve zebrafish performance in a binary discrimination task, by increasing the cost associated with incorrect responses. In fact, in the longitudinal apparatus, the distance between the two doors was much larger than in the square apparatus. Thus, a fish that emitted a wrong choice, had then to swim much longer to reach the correct tunnel on the opposite side of the corridor.

Based on this result, one might argue that the distance between the two stimuli in the I-maze might have actually partially impaired the discrimination ability of zebrafish. In fact, in this set-up, once a fish approached one of the two stimuli, it found itself at a considerable distance from the other stimulus, whose retinal projection thus became relatively small. It has been shown that perceptual salience (i.e., size) of a stimulus affects discrimination performance in zebrafish [39] (but see [46] for evidence of successful discrimination with smaller stimuli). The distance from the stimuli could theoretically have actually made it more difficult for the zebrafish to correct initial mistakes, if the first stimulus approached would not be the correct one. This could explain why the difference between the two test apparatuses might emerge only for the total number of choices, but not for the first choices. Moreover, the effect of distance from stimuli might be more pronounced when the fish are faced with the unfamiliar test stimuli, which are more challenging to process than the familiar training images. This could explain why this effect emerged only during the amodal completion test. However, we believe this explanation to be unlikely. In fact, in a previous study, using an I-maze of the same length as ours and smaller stimuli, zebrafish were able to discriminate a familiar from an unfamiliar visual stimulus [47].

Last but not least, the last two experiments provided the first demonstration that amodal completion can be observed in zebrafish. Indeed, in both Exp. 3 and 4, fish reinforced on the full disk preferred the figure in which the occluder was perfectly juxtaposed to the amputated disk. In human observers, this display gives the impression of representing a full disk partially hidden behind the black-green occluder (amodally completing behind it). Conversely, fish reinforced on the amputated disk preferred the configuration in which a white space was left between the occluder and the amputated disk, so preventing amodal completion. This is the first demonstration of amodal completion in zebrafish. While other fish species already showed their sensitivity to such an illusory percept (redtail splitfins, [19]; coral reef fish [20]; sharks [21]), this crucial phenomenon had never been investigated in zebrafish before. The current results thus add to the extant evidence that amodal completion mechanisms are widespread among fish species as well, despite the great taxonomic diversity of this clade. Together with the results previously obtained in other vertebrate and invertebrate species [12–18], our finding stresses the ubiquitous nature of amodal completion mechanisms among animal species. Moreover, given the importance of zebrafish as a model for the study of the visual system, our current results pave the way for further investigations on the neurobiology of amodal completion. For instance, future studies could capitalise on our results by identifying the brain areas recruited in amodal completion in zebrafish. By applying the wide range of molecular and genetic tools available in this species (see [1–5]), this could allow to explore the neurobiology of the mechanisms supporting amodal completion to a degree that has been impossible so far.

## Supporting information

S1 Data (XLSX)

S2 Data (XLSX)

S3 Data (XLSX)
